# Ubiquitin‐specific peptidase 53 inhibits the occurrence and development of clear cell renal cell carcinoma through NF‐κB pathway inactivation

**DOI:** 10.1002/cam4.3911

**Published:** 2021-05-11

**Authors:** Dingwen Gui, Zhufeng Dong, Wei Peng, Weidong Jiang, Geng Huang, Gang Liu, Zhihua Ye, Yang Wang, Zuwei Xu, Jinlun Fu, Shuai Luo, Yunfei Zhao

**Affiliations:** ^1^ Department of Urology Huangshi Central Hospital (Affiliated Hospital of Hubei Polytechnic University) Edong Healthcare Group Huangshi P.R. China; ^2^ Hubei Province Key Laboratory of Occupational Hazard Identification and Control Wuhan University of Science and Technology Wuhan P.R. China; ^3^ Wuhan University School of Basic Medical Sciences Wuhan P.R. China

**Keywords:** clear cell renal cell carcinoma, NF‐κB pathway, RNA sequencing, USP53

## Abstract

**Background:**

Clear cell renal cell carcinoma (ccRCC) is one of the most prevalent malignant diseases in the urinary system with more than 140,000 related deaths annually. Ubiquitination–deubiquitination homeostasis is an important factor in ccRCC progression; ubiquitin‐specific peptidase 53 (USP53) belongs to the family of deubiquitinating enzymes, but its functions are rarely reported.

**Methods:**

Databases obtained from GEO and TCGA were analyzed to reveal the role of USP53 in ccRCC. CCK‐8/BrdU and EDU assays were used to detect the proliferation of ccRCC after USP53 overexpression or knockdown. A tumor xenograft experiment was used to verify the effect of the proliferation of ccRCC after USP53 knockdown. Transwell assays were used to detect the metastasis of ccRCC after USP53 overexpression or knockdown. RNA sequencing and western blot analysis were employed to detect the change in genes after USP53 overexpression and knockdown. Then we tested the effect of USP53 on IκBα protein stability through western blot analysis. Detect the effect of USP53 on IκBα ubiquitination in vitro by immunoprecipitation method.

**Results:**

USP53 expression was downregulated in ccRCC tissues and USP53 expression was significantly negatively correlated with the tumor progression and clinical prognosis. The ability of growth and metastasis of ccRCC was inhibited after USP53 overexpression. In addition, USP53 knockdown promoted ccRCC growth and metastasis. Moreover, USP53 knockdown promoted the ability of clone formation of ccRCC *in vivo*. NF‐κB signaling pathway significantly enriched and downregulated in USP53 overexpressed cells, and genes in the NF‐κB pathway (such as IL1B, CXCL1‐3, RELA, RELB, etc.) were obviously downregulated in USP53 overexpressed cells. USP53 overexpression decreased the phosphorylation of IKKβ and P65 in both Caki‐1 and 786‐O cells, and the expression of IκBα was increased. Phosphorylation of IKKβ and P65 was increased in both Caki‐1 and 786‐O cells after USP53 knockdown. As the expression of USP53 increases, the protein expression of IκBα was also gradually increased and USP53 reduced the ubiquitination of IκBα.

**Conclusion:**

In summary, our data indicate that USP53 inhibits the inactivation of the NF‐κB pathway by reducing the ubiquitination of IκBα to further inhibit ccRCC proliferation and metastasis. These findings may help understand the pathogenesis of ccRCC and introduce new potential therapeutic targets for kidney cancer patients.

## Graphical abstract

Our data showed that USP53 inhibits ccRCC proliferation and metastasis through NF‐κB pathway inactivation. These findings may help understand the pathogenesis of ccRCC and introduce new potential therapeutic targets for kidney cancer patients.

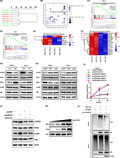

## 1 INTRODUCTION

Renal cell carcinoma (RCC) accounting for about 90% of kidney cancers, is the third most common urological cancer after prostate and bladder cancer but has the highest mortality rate at about 25%.^1^, ^2^, ^3^ According to recent pathological classification by the International Society of Urological Pathology (ISUP), RCC mainly includes clear cell (ccRCC), papillary (pRCC), and chromophobe (chRCC) subtypes,^4^ with ccRCC being the most common subtype.^5^ ccRCC carries a poor prognosis usually appearing in adult patients around their 60 years old and its pathogenesis is not well understood.^6^, ^7^ The patients with ccRCC are not easily detectable in the early stages, and the main treatments are surgical removal of lesions and combined chemotherapy or biotherapy.^8^ Metastasis is the primary cause of tumor death in ccRCC,^9^, ^10^ thus improved diagnostic markers of metastasis are urgently needed in diagnosis and treatment of ccRCC.^11^, ^12^ Although surgical treatment has achieved some clinical effects, the 5‐year survival rate of patients with late‐stage or metastasis of kidney cancer is still low.^13^, ^14^ Nearly 30% of patients with localized RCC have recurrence and metastasis after tumor resection.^15^, ^16^ The root cause of ccRCC difficult to diagnose and cure is that the pathogenesis and molecular mechanism of ccRCC are not clear.^17^ It is particularly important to study the molecular mechanism of ccRCC and to seek safe and effective biological targets.

In recent years, ubiquitination–deubiquitination is increasingly being found to play a major role in the formation of malignant tumors.^18^ Ubiquitination–deubipuitination mediated multi‐tumor occurrence and development through post‐translational modifications, in which ubiquitination–deubipuitination homeostasis is an important factor in maintaining ccRCC progression.^19^, ^20^, ^21^, ^22^ Currently, deubiquitinating enzymes can be divided into six major families five of which are serine proteases, namely ubiquitin‐specific protease family (USPs), ubiquitin carboxy terminal hydrolase (UCHs), MJD domain protease family (MJDs), ovarian tumor‐associated protease family (OTUs), and herpesvirus tegument USPs (htUSPs).^23^, ^24^ Deubiquitinating enzymes regulate multiple signaling pathways related to cancer, such as EGFR, NF‐κB, TGF‐β, and the like.^25^, ^26^ Deubiquitinating enzymes can also affect cancer progression through the cell cycle, migration, apoptosis, and DNA damage during cancer development.^19^, ^27^ Among the six deubiquitinating enzyme families, the study of USPs is relatively clear. USPs such as USP2a, USP4, and USP7 can affect the progression of cancer by regulating the stability of p53 protein.^28^ USP53 belongs to USPs family, but there have been no reports of its involvement in cancer, so we want to explore the effect of USP53 on the development of kidney cancer.

In this study, we conducted a systematic study to identify the correlation between the malignant degree of renal clear cell carcinoma and gene expression. The expression level of USP53 gene decreases with the increase in the malignant degree of renal clear cell carcinoma, and the patient has a worse prognosis. We overexpress and knockdown ubiquitin‐specific protease 53 (USP53) in the ccRCC cell lines Caki‐1 and 786‐O. The ability of these two cells to proliferate and migrate *in vitro* is then examined. Next, the ability of tumor formation under in vivo conditions after USP53 knockdown was studied by subcutaneous tumor formation in nude mice. Combining RNA sequencing assay with bioinformatics algorithms, we found USP53 regulates proliferation and migration of ccRCC cells via NF‐κB pathway.

These studies show USP53 may be an important target for gene therapy of kidney cancer.

## 2 MATERIALS AND METHODS

### 2.1 Data sources

GSE66271, GSE76207, GSE36895, and GSE26574 were obtained from the Gene Expression Omnibus (GEO) database (http://www.ncbi.nlm.nih.gov/geo/). Level 3 mRNA‐seq data of kidney renal clear cell carcinoma (KIRC), including 539 tumor samples and 72 adjacent normal samples, were downloaded from TCGA database (http://www.cbioportal.org/). Clinical information, including tumor stage, survival time, and outcome, was also extracted from TCGA.

### 2.2 Expression analysis

The KIRC RNA‐seq level 3 and GSE76207 count data were normalized with the negative binomial distribution methodology by DESeq2. GSE66271, GSE36895, and GSE26574 were examined by affymetrix array, the raw data analyzed by affy R package. KIRC tumor samples were divided into various groups based on tumor stage. Then differential expression analysis of the tumor samples in each group was conducted by *t*‐test.

### 2.3 Survival analysis

Patients with high and low expression levels based on USP53 expression media values. If the USP53 expression level in a patient was higher than the media value, the patient was classified as having a high expression level; otherwise, the patient was classified as having a low expression level. The log‐rank test was used to calculate the significance of survival time differences between the two classes of patients.

### 2.4 Cell lines

Two ccRCC cell lines (786‐O and Caki‐1), human embryonic kidney 293T were purchased from the American Type Culture Collection and used for no more than 6 months after resuscitation. 786‐O and HEK‐293T were cultured in a high glucose DMEM medium (Gibco) supplemented with 10% fetal calf serum (Bio‐One) and 1% Penicillin and Streptomycin (Gibco), Caki‐1 was cultured in McCoy's 5a medium supplemented with 10% fetal calf serum (Bio‐One) and 1% Penicillin and Streptomycin (Gibco). All cells were maintained at 37°C in a sterile incubator (Esco) containing 5% CO_2_.

### 2.5 Lentivirus production and infection

The human full‐length USP53 was amplified from human complementary DNA (cDNA) and cloned into the pHAGE‐flag vector. Two shRNA for USP53 were designed, and the target sequences were constructed into the pLKO.1 vector. The plasmids were transfected into HEK‐293T cells using PEI transfection reagent (Sigma, #GF95977287), along with psPAX2 and pMD2.G helper plasmids. The virus supernatant was added to the cell culture medium in the presence of 10 μg/ml polybrene. Positive clones were obtained upon puromycin selection and then detected by real‐time PCR or western blotting assay.

### 2.6 Cell Counting Kit‐8 assays

For cell counting kit‐8 (CCK‐8, bimake, B34304) assay, USP53 overexpression and knockdown cells were seeded with 100 μl of a 96‐well plate at a cell density of 3 × 10^4^/ml. Then, the cells were incubated with CCK‐8 regent for 3 h twice a day. The absorbance was measured at 450 nm using an enzyme‐linked immunoassay (Thermo, multiskan MK3). The sample is made of three attached wells.

### 2.7 BrdU assay

A BrdU assay was performed using the BrdU Cell Proliferation Assay Kit (Roche, 11647229001) according to the manufacturer's instructions. Briefly, cells were pulsed with 10 μM of BrdU for 4 h and fixed. After denaturation of the genomic DNA, cells were incubated with an anti‐BrdU‐peroxidase antibody for 90 min. Then, the substrate tetramethyl‐benzidine was added, and the colored reaction product was quantified by spectrophotometry.

### 2.8 EDU Immunofluorescence Staining

Cells were seed 200 μl in a 48‐well plate at a density of 1.5×10^5^/ml. The cells were incubated 2 h by EDU marketing solution (Ribobio, #C10310‐1), and then fixed by 4% paraformaldehyde for 30 min, and the stain solution was added in the plate for 30 min. Following three washes with PBS, cells were counterstained with Hoechst (dilution 1:1000; Ribobio, # C10310‐1) for 10 min at room temperature for nuclear staining. Then observe the photo under a fluorescence microscope (Olympus, IX73).

### 2.9 Wound healing assay

Cells were infected with lentivirus as described above and seed in 6‐well plates. Once the cells reached 100% confluence, a linear scratch wound was created using a 200 µl pipette tip. The cells were washed twice with PBS to remove detached cells. Then, the cells were incubated at 37 °C in an atmosphere containing 5% CO_2_, and the wounded area was monitored using a microscope (Olympus IX73) and measured using an Adobe Illustrator CC 2017 (NIH).

### 2.10 Transwell assays

Cell migration assays were performed on 24‐well Transwell cell culture plates (Corning, #3421) with a polycarbonate membrane at the bottom of the upper chamber with 5 μm pores. Cells were seed in the upper chamber cultured with 100 μl medium without FBS, and then 600 μl medium containing 10% FBS add into the lower chamber. For the cell invasive assays, the upper chamber of the Transwell was coated with Matrigel (BD, #354234). Experiments were performed a similar to the cell migration assays. At the indicated time points, the non‐migrated cells on the upper surface were removed by wiping with a cotton swab, and the migrated cells on the lower surface were fixed with 4% paraformaldehyde for 15 min, dried in a ventilated place, and stained with 0.1% crystal violet for 30 min, randomly select five fields under the microscope (Olympus, IX73) to count the number of cells, and perform three independent experiments.

### 2.11 Western blotting analysis

Protein was extracted using lysis buffer (RIPA, 1% TritonX‐100, 0.1% sodium dodecyl sulfate, 1% sodium deoxycholate, 0.15 M NaCl, and 10 mM Tris, pH 7.2). The protein concentration was assessed using a Pierce BCA Protein Assay Kit (Thermo, #23225). Protein samples were separated by electrophoresis on a 12.5% sodium dodecyl sulfate–polyacrylamide gel and transferred to a nitro membrane. The specific antibody for detecting the protein was prepared with 5% milk and incubated at 4°C overnight, and then the membrane was incubated for 1 h at room temperature in an anti‐host protein horseradish peroxidase conjugate, and then the immunoreactive band was observed by Femto ECL substrates (Thermo Fisher Scientific) and visualized using a ChemiDoc MP Imaging System (Bio‐Rad).

### 2.12 RNA extraction and quantitative RT‐PCR

Total RNA was extracted using Trizol (Roche). Reverse transcription of total RNA (2 μg) with a Revert Aid First Strand cDNA synthesis kit (Roche) according to the manufacturer's instructions. Β‐actin was selected as the internal control. The relative expression (RQ) was calculated as the fold change relative to an internal reference, which was based on the following equation: RQ =  2^−ΔΔCt^.

The primer sequences for USP53 were as follows: forward 5′‐GCCTAAATGCA AACAAAGTTGC‐3′ and reverse 5′‐TTTGTTCAGAAGGGCAGCTTGA‐3′. The primer sequences for the housekeeping gene β‐actin were as follows: forward 5′‐CATGTACGTTGCTATCCAGGC‐3′ and reverse 5′‐CTCCTTAATGTCACGCACG AT‐3′.

### 2.13 RNA‐seq assay

For a RNA‐seq assay, cDNA libraries were constructed, and single‐end libraries were sequenced by BGISEQ 500. HISAT2 software (version 2.21) was used to align clean reads to human genomes. Then, SAMtools (version 1.4) was stored and converted aligned reads to Binary Alignment Map format. The fragments per kilobase per million and reads counts of each identified gene were calculated by String Tie (version 1.3.3b). Next, DESeq2 was used to analyze differential expressed genes (DEGs). DEGs were screened through two criteria: (a) a fold change larger than 1.5 and (b) a corresponding adjusted P value less than 0.05.

The primer sequences for USP53 were as follows: forward 5′‐TCGGGTTTAAACG GATCCATGGCATGGGTAAAATTCTTAC‐3′ and reverse 5′‐GGGCCCTCTAGAC TCGAGCTAAGATAGTGAATTATTACAAAAGCCA‐3′.

### 2.14 Tumor formation in nude mice

Female BALB/c nude mice (age, 6–8 weeks old, body weight, and 20–25 g) were purchased from Beijing Vital River Laboratory Animal Technology Co., Ltd. (Beijing, China) and housed under specific pathogen‐free conditions with free access to food and water in the animal facility of Wuhan University. All animal studies were carried out in accordance with the Animal Care and Use Committee of Wuhan University, which is consistent with the Association for Assessment and Accreditation of Laboratory Animal Care international (AAALAS) guidelines.

To assess the effect of USP53 on ccRCC growth, USP53 knockdown Caki‐1 cells and respective control cells were subcutaneously injected into the bilateral scapula of BALB/c nude mice (3 × 10^6^ cells in 0.3 ml Matrigel per mouse, eight mice per group). Tumor growth was monitored by measuring the tumor volumes every week. After 11 weeks, all mice were sacrificed and photographed. Tumor tissues were stripped carefully.

### 2.15 Immunohistochemistry

Immunohistochemistry of USP53 was performed on paraffin‐embedded tissue sections using USP53 (1:50, A14353, Abclonal) antibody. Briefly, samples were heated for 20 min in EDTA buffer then placed in 3% H_2_O_2_ for 20 min to quench endogenous peroxide activity. After washing with PBS, slides were blocked with 10% BSA for 10 min and incubated with the primary antibodies overnight at 4°C, slides were incubated with enhanced enzyme‐labeled goat anti‐rabbit IgG (Beijing ZSGB Biotech) for 1 h at room temperature. Immunohistochemical staining was visualized using 3,30‐diaminobenzidine (DAB) substrate kit (Beijing ZSGB Biotech) and further stained with hematoxylin. Images were captured on a light microscope (Olympus).

### 2.16 Statistical analysis

Experimental data were managed and analyzed by Graph Pad Prism 5.0 and represented as the mean ± SEM for at last three separate experiments. Paired *t*‐tests were used for two‐group comparisons, *p* < 0.05 was considered statistically significant.

## 3 RESULTS

### 3.1 Reduced expression of USP53 in ccRCC

**FIGURE 1:**
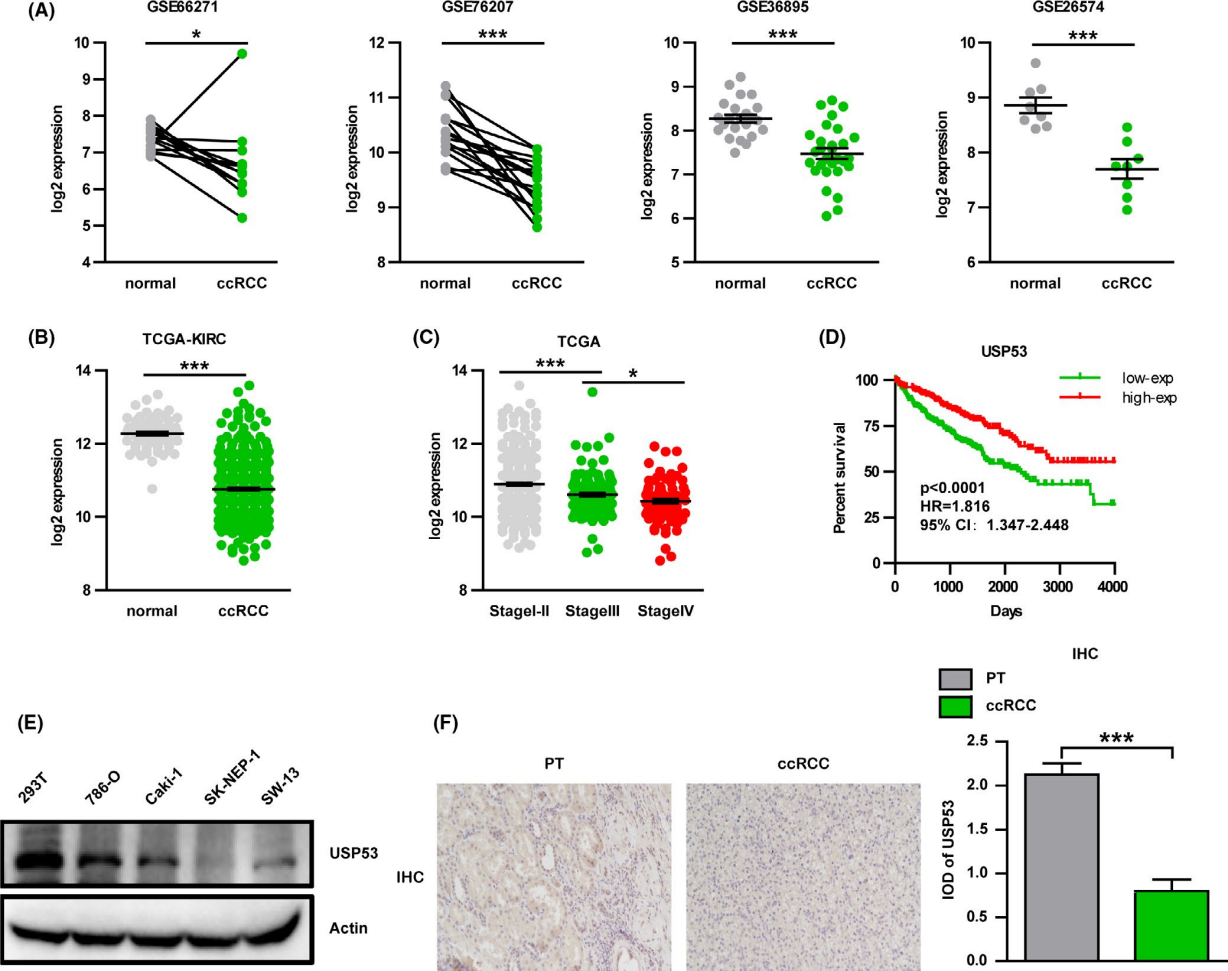
Reduced expression of USP53 in renal cell carcinoma. (A) GES database (GES66271/GES76207/GES36895/GES26574) analysis showed compared with normal tissues; the expression of USP53 in ccRCC tissues was significantly reduced. (B) TCGA‐KIRC database analysis shows the expression of USP53 in ccRCC tissues was significantly reduced. (C) Low expression of USP53 is positively correlated with tumor grade. (D) Low expression of USP53 is positively correlated with poor prognosis. (E) Western blot assays show that the expression of USP53 in tumor cell lines (786‐O, Caki‐1, SK‐NEP‐1, SW‐13) is significantly reduced compared with normal cell lines (293T). (F) Immunohistochemical staining shoe that the expression of USP53 in tumor tissues was significantly lower than paracancerous tissues. (*p* < 0.0001, HR = 1.816, 59% CI: 1.347‐2.448)

We used bioinformatics to analyze the differential gene analysis of ccRCC through clinical sample information and found that USP53 was significantly related to CCRCC. Analysis results of four GEO databases (GSE66271, GSE76207, GSE36895, and GSE26574) showed that expression of USP53 in ccRCC significantly lower than normal tissue (Figure 1A). We further analyzed the expression of USP53 in TCGA‐KIRC database, consistent with GEO's results, the expression of USP53 in ccRCC is reduced (Figure 1B).

In‐depth analysis of the clinical significance of USP53 expression and tumor stages, ccRCC samples were divided into I‐II, III, and IV stages, the results showed that as tumor grade increases, USP53 expression decreases (Figure 1C). Moreover, according to the expression of USP53, ccRCC samples were classified into USP53 high expression population and USP53 low expression population and used for survival analysis. The Kaplan–Meier curve showed the low expression of USP53 have poor overall survival (Figure 1D). We also tested the expression of USP53 in human normal renal epithelial cells (293) and four renal cancer cell lines (RCC) by RT‐PCR and western blot. The results showed that compared with normal kidney cells, USP53 is significantly lower expressed in kidney cancer cells. These data also suggest that USP53 may act as a tumor suppressor gene in kidney cancer (Figure 1E). Then, we performed immunohistochemical staining on the tissues of patients with renal clear cell carcinoma and found that the expression of USP53 in tumor tissues was significantly lower than paracancerous tissues (Figure 1F).

### 3.2 Overexpression USP53 inhibits the growth and proliferation of ccRCC

**FIGURE 2:**
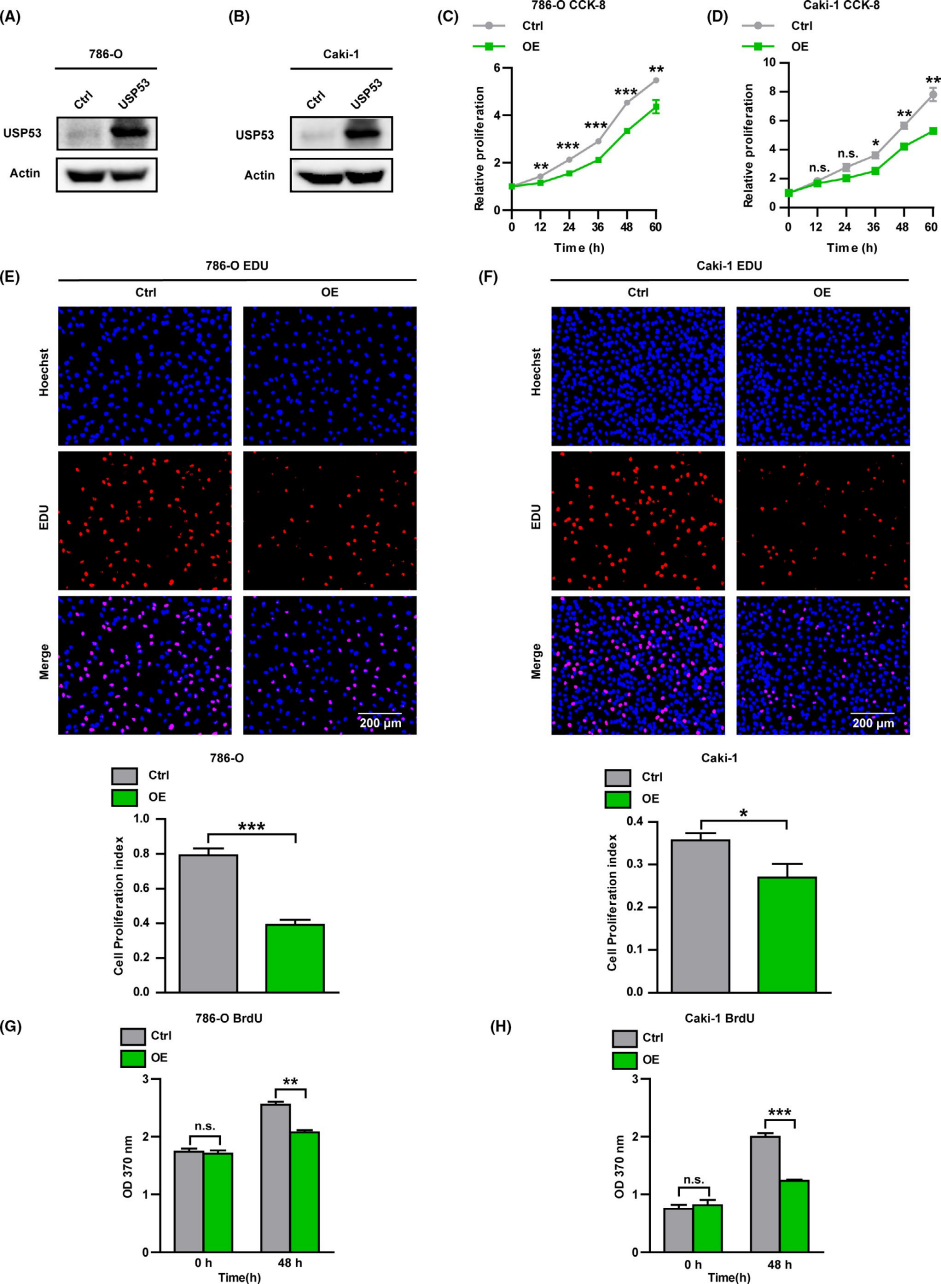
Overexpression USP53 inhibits the growth and proliferation of ccRCC. (A, B) Immunoblotting assays were analyzed to determine the protein expression of USP53 in control and USP53 overexpression cells (786‐O‐USP53‐OE/Caki‐1‐USP53‐OE) or control cells (Ctrl). (C, D) Cell proliferation is impaired by overexpression in 786‐O and Caki‐1 cells, as assessed by CCK‐8 assay at the indicated time points. (E, F) EDU cell proliferation assay demonstrates the inhibitory effect of USP53 overexpression on both 786‐O and Caki‐1 cells. Cell proliferation index of 786‐O and Caki‐1 USP53 expression and control were statistically analyzed (bottom). (G, H) BrdU cell proliferation assays reflect 786‐O and Caki‐1 cells that overexpress the USP53 gene proliferation ability was significantly inhibited at 48 h after inoculation after USP53 overexpression. Cell numbers were counted by Image pro plus software and are shown at right (*p < 0.05, **p < 0.01, ***p < 0.001. Data are shown as mean ± SEM)

To test the effect of USP53 on proliferation in ccRCC, we inserted the USP53 gene into a plasmid vector, packaged it into a lentivirus, and constructed a cell line stably expressing USP53 by infection with Caki‐1 and 786‐O. USP53 protein expression was significantly increased in a stable‐transfected cell lines by western‐blot assay (Figure 2A,B). Through CCK‐8 assay, we found that overexpression of USP53 in Caki‐1 and 786‐O cells significantly inhibited cell proliferation (Figure 2C,D). Then EDU cell proliferation imaging assay visually reflects that the proliferation of ccRCC 786‐O and Caki‐1 is significantly inhibited after USP53 overexpression (Figure 2E,F). BrdU cell proliferation assay was also used to test the proliferative ability of cells, and the proliferation of USP53 overexpression cells was inhibited compared with control (Figure 2G,H).

### 3.3 Knockdown USP53 promotes the growth and proliferation of ccRCC

**FIGURE 3:**
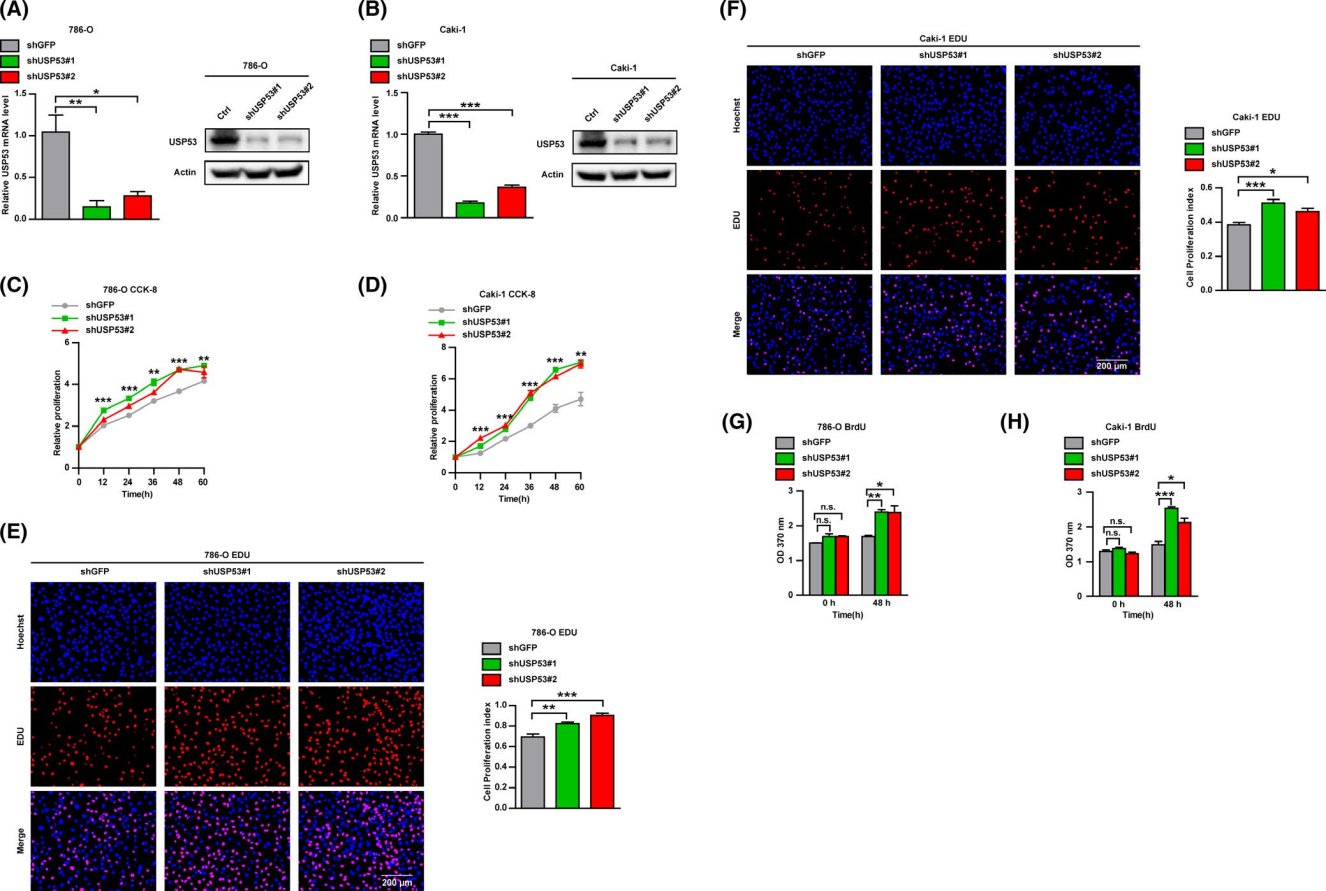
Knockdown USP53 promotes the growth and proliferation of ccRCC. (A, B) mRNA levels of USP53 in Caki‐1 and 786‐O cells were analyzed by real‐time PCR after infection with Lentivirus targeting USP53 (USP53 shRNA) or negative controls (Ctrl). (C, D) Cell proliferation of the USP53 depleted or control cells was evaluated by CCK8 assays at the indicated time points. (E, F) EDU cell proliferation immunofluorescence assay were executed to determine the proliferation and viability of the USP53 depleted or control cells, Scale bar, 200 μm. Cell proliferation index of USP53 shRNA and control shRNA in 786‐O or Caki‐1 cells were statistically analyzed (right panel). (G, H) BrdU cell proliferation assay were executed to determine the growth and viability of the USP53 depleted or control cells. Representative images and quantification are shown (*p < 0.05, **p < 0.01, ***p < 0.001). Data are shown as mean ± SEM

To further explore the biological function of USP53 in ccRCC development, shRNA‐mediated loss‐of‐function of USP53 in 786‐O and Caki‐1 cells was used to evaluate the critical role of USP53 in cell growth and proliferation. Four independent shRNA clones were introduced into the Caki‐1 cells and 786‐O cells to evaluate the functional role of USP53 in cell growth and proliferation. Among them, two shRNA‐mediated knockdown of USP53 gene has high specificity and efficiency, and shGFP as control (Figure 3A,B). The ability of cell growth and proliferation was tested by CCK‐8, EDU, and BrdU cell proliferation assays. We found that the growth and proliferation of USP53 knockdown cells were significantly promoted compare with shGFP (Figure 3C,D). EDU cell proliferation imaging assay and BrdU cell proliferation assay also showed that the proliferation of ccRCC after USP53 knockdown was promoted compare with shGFP (Figure 3E–H).

### 3.4 Overexpression USP53 inhibits migration and invasion of ccRCC

**FIGURE 4:**
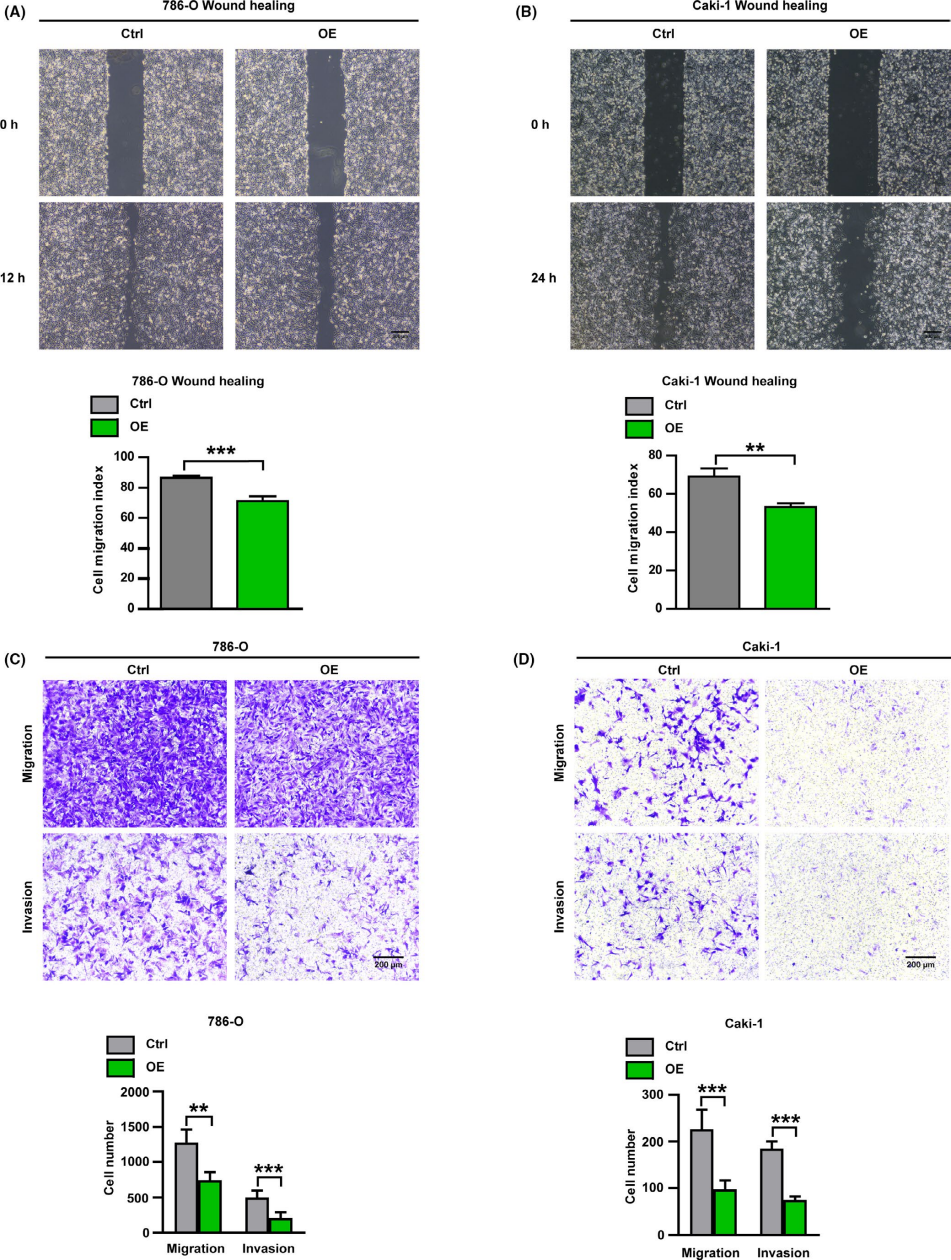
Overexpression USP53 significantly inhibits migration and invasion of ccRCC. (A, B) Wound healing assays were used to evaluate the cell migration in USP53 overexpression or control cells. Representative images of USP53 overexpression cells or control cells in 786‐O and Caki‐1 cells in indicated time points were shown. Scale bar, 300 μm. Cell migration indexes were quantified at the bottom. (C, D) Cell migration and invasion assays were performed in Transwell assays with or without Matrigel, and USP53 overexpression significantly inhibits the cell migration and invasion. (C) Transwell assays in 786‐O cells with overexpressed USP53 or controls were determined at 10 h by taking images of the migrated cells, Scale bar, 200 μm. (D)Transwell assays in Caki‐1 cells with overexpressed USP53 or controls were determined at 24 h by taking images of the migrated cells, Scale bar, 200 μm. Representative photographs and the migration index are shown at right

Cell migration and invasion are vital hallmarks of tumor cells, especially RCC.^12^, ^29^ Metastasis is an important factor in the high mortality rate of ccRCC. Figures 2 and 3 showed that USP53 significantly affected cell proliferation in ccRCC; we hypothesized that if USP53 could have an effect on the migration or invasion of the ccRCC cell lines. We first examined the repair ability of Caki‐1 and 786‐O cells by wound healing assays; results showed that overexpression of USP53 significantly inhibited the ability of wound healing repair at 12 and 36 h for 786‐O and Caki‐1 (Figure 4A,B). The ability of cell migration and invasion was tested via Transwell assays, we found fewer migrated cells on the Transwell chamber surface compared to the controls after overexpression USP53 (Figure 4C,D), the quantitative results are at the bottom of the picture.

### 3.5 Knockdown USP53 promotes migration and invasion of ccRCC

**FIGURE 5:**
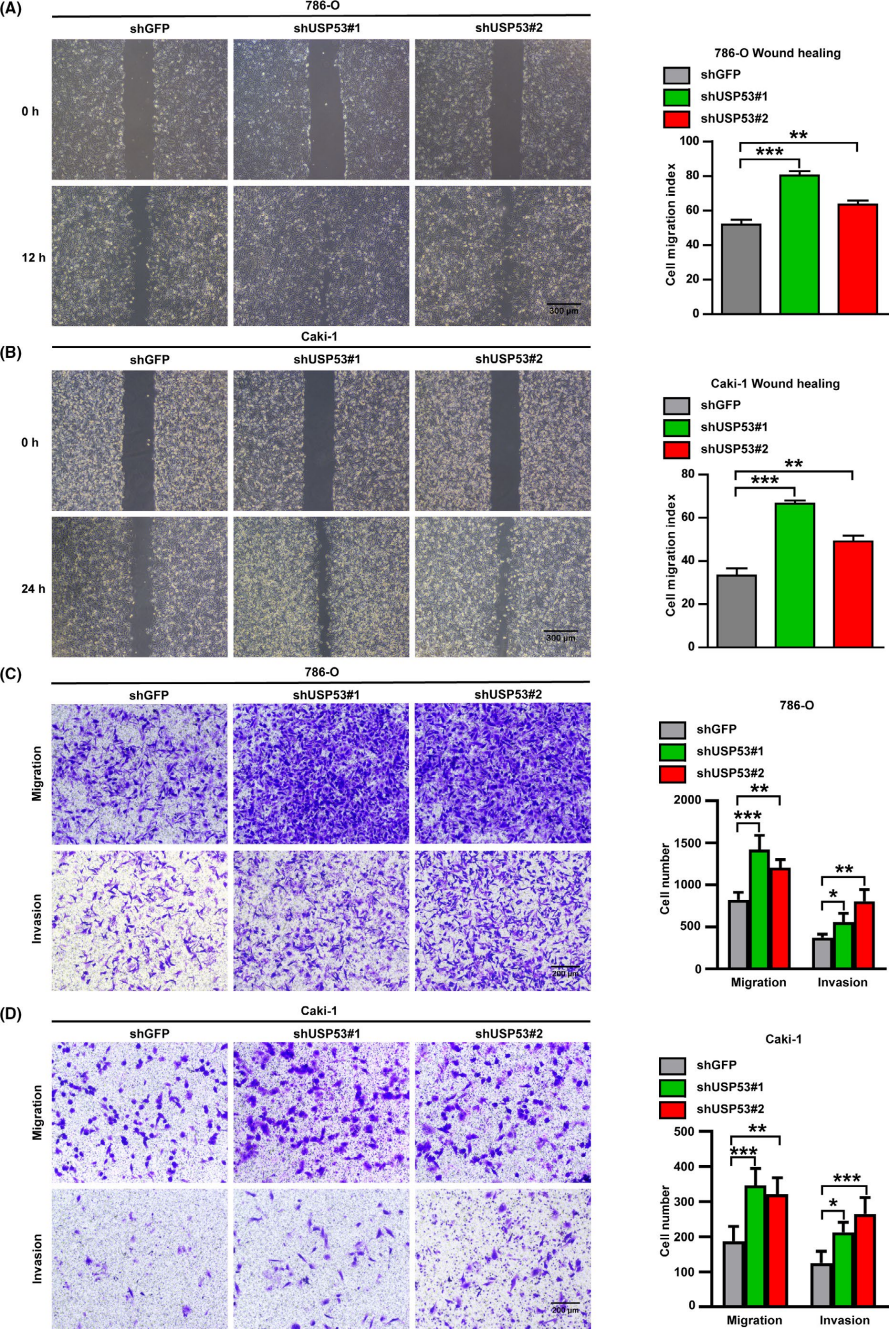
Knockdown USP53 significantly promotes migration and invasion of ccRCC. (A, B) Cell wound healing ability was promoted at indicated time points after USP53 depleted compare with control through wound healing assay. In 786‐O cells (A) Caki‐1 cells (B). Scale bar 300 μm. Relative cell migratory distance of Caki‐1 USP53 overexpression and Control were quantified and statistically analyzed (right panel). (C, D) Transwell migration and invasion assays were performed in Transwell assays with or without Matrigel, and USP53 knockdown significantly inhibits the cell migration and invasion. (C) Representative Images of Transwell migration assay of USP53 depleted in 786‐O cells and Control cells for 10 h. Scale bar, 200 μm. (D) Representative images of Transwell invasion assay of USP53 depleted in Caki‐1 cells and Control cells for 24 h. Scale bar, 200 μm

Similarly, we examined the effect of USP53 knockdown on the metastatic and invasive ability of ccRCC. We test repair ability of 786‐O and Caki‐1 after knockdown USP53. The repair ability of knockdown cells at 12 hours was significantly stronger than that of the control group in 786‐O (Figure 5A). In addition, Caki‐1 that knockdown USP53 has a stronger ability to repair at 24 h compared with control cells after the scratches (Figure 5B). We then performed the same migration and invasion assay with shRNA knockdown on USP53 cell lines. The results showed that USP53 knockdown significantly promoted the migration and invasion of renal cancer cells Caki‐1 and 786‐O cells (Figure 5C,D). Therefore, it can be explained that the gene USP53 can inhibit the migration and invasion of renal cancer cells. The quantitative results are at the right of the picture.

### 3.6 NF‐κB pathway was involved in the ccRCC inhibitory effect of overexpression USP53

**FIGURE 6:**
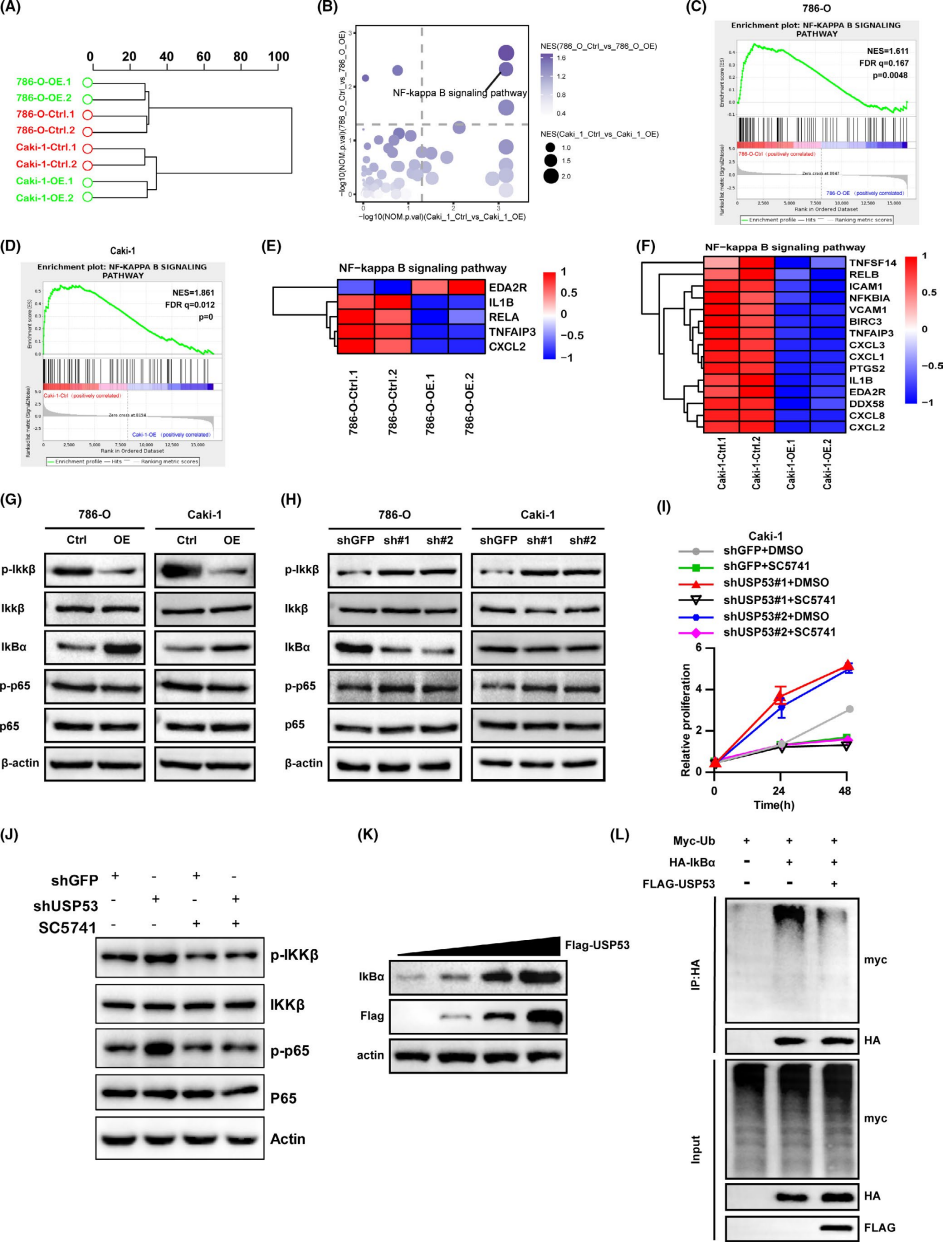
NF‐κB pathway was changed in ccRCC cell lines after USP53 overexpression. (A) Hierarchical clustering analysis of RNA sequencing data revealed significant differences in gene expression between USP53 overexpressing cells and control cells. (B) GESA analysis showed that the NF‐κB pathway was changed significantly after USP53 overexpression in 786‐O and Caki‐1 cells. (C, D) Enrichment of an NF‐κB‐dependent gene expression signature in GSEA analysis of genes altered as described above. (E, F) Heat map representation of the differential expression of NF‐κB‐related genes between overexpressed and control cells. (G, H) Phosphorylation of IKKβ and p65 were estimated by Western blotting assay in USP53 overexpressed cells (G) or depletion cells (H). (I, J) Rescue experiments showed that USP53 inhibits the proliferation of Caki‐1 cell line by inhibiting the NF‐κB pathway. (K) The expression level of IκBα increased with the increase in USP53 expression in 293 cells; (L) USP53 can significantly reduce the ubiquitination of IκBα in 293 cells

As USP53 plays essential roles in cell proliferation, migration, and invasion in ccRCC, it is necessary to clarify the involved mechanisms of USP53. RNA sequence analysis was performed in Caki‐1 and 786‐O cells overexpressing USP53. Principal component analysis (PCA) showed significant differences in gene expression between USP53 overexpressing cells and control cells (Figure 6A). We performed GESA analysis on the RNA sequencing results of 786‐O and Caki‐1 cells overexpressing USP53. The results showed that the NF‐κB pathway was changed significantly after USP53 overexpression in 786‐O and Caki‐1 cells (Figure 6B). Gene‐set enrichment analysis (GSEA) indicates that NF‐κB signaling pathway significantly enriched and downregulated in both 786‐O and Caki‐1 USP53 overexpressed cells (Figure 6C,D). Significantly, genes in the NF‐κB pathway (such as IL1B, CXCL1‐3, RELA, RELB, etc.) were obviously downregulated in both 786‐O and Caki‐1 USP53 overexpressed cells (Figure 6E,F). Among these differential genes, most inflammation‐related genes positively regulate cell survival, which supports the functional roles of USP53. To further determine if USP53 mediate cell growth and migration inhibition through NF‐κB signaling pathway, we analyzed the phosphorylation of IKKβ, P65, and IκBα in USP53 overexpression and knockdown cells via immunoblot assays. The result showed that USP53 overexpression decreased the phosphorylation of IKKβ and P65 in both Caki‐1 and 786‐O cells. The expression of IκBα was increased, which was essential in the regulation of NF‐κB activity (Figure 6G). Phosphorylation of IKKβ and P65 were increased in both Caki‐1 and 786‐O cells after USP53 knockdown (Figure 6H).

To detect that USP53 indeed acts through that pathway, we used USP53 knockdown Caki‐1 cells for cell growth experiments and treated the cells with NF‐κB inhibitor SC5741 as a control group. The results showed that the growth ability of shUSP53#1+DMSO and shUSP53#2+DMSO groups was significantly enhanced. The growth ability of shUSP53#1+SC5741 and shUSP53#2+ SC5741 groups was similar to that of shGFP+SC5741 group, and was significantly lower than that of shUSP53#1+DMSO and shUSP53#2+DMSO groups (Figure 6I). The WB experiment was further used to detect the protein expression of the cells after the NF‐κB inhibitor SC5741 was incubated with the cells. The results showed that the protein expression levels of p‐IKKβ and p‐p65 in the shUSP53 group were significantly increased compared with the shGFP group. After SC5741 incubated the cells, the expression of p‐IKKβ and p‐p65 in the shUSP53 group and shGFP group did not change significantly (Figure 6J).

The P65 and P50 complexes are inhibited by IκBα binding and cannot enter the nucleus to regulate gene transcription. In order to further explore the influence of USP53 on the expression and stability of IκBα, we transfected USP53 in different amounts in four groups of 293 cells, and then detected the changes in IκBα protein expression. The results showed that the expression of USP53 and IκBα in 293 cells has the same trend (Figure 6K). In order to figure out why USP53 will affect the expression of IκBα, we transfected Myc‐ub, HA‐IκBα, and Flag‐USP53 into 293 cells, and then tested the effect of USP53 on the ubiquitination of IκBα by co‐immunoprecipitation and WB experiments. The results showed that USP53 can significantly reduce the ubiquitination of IκBα (Figure 6L). In summary, USP53 inhibits the p65 and p50 complex by deubiquitinating IκBα, and further inhibits the activity of the NF‐κB pathway.

### 3.7 Knockdown USP53 promotes the growth of ccRCC *in vivo*

**FIGURE 7:**
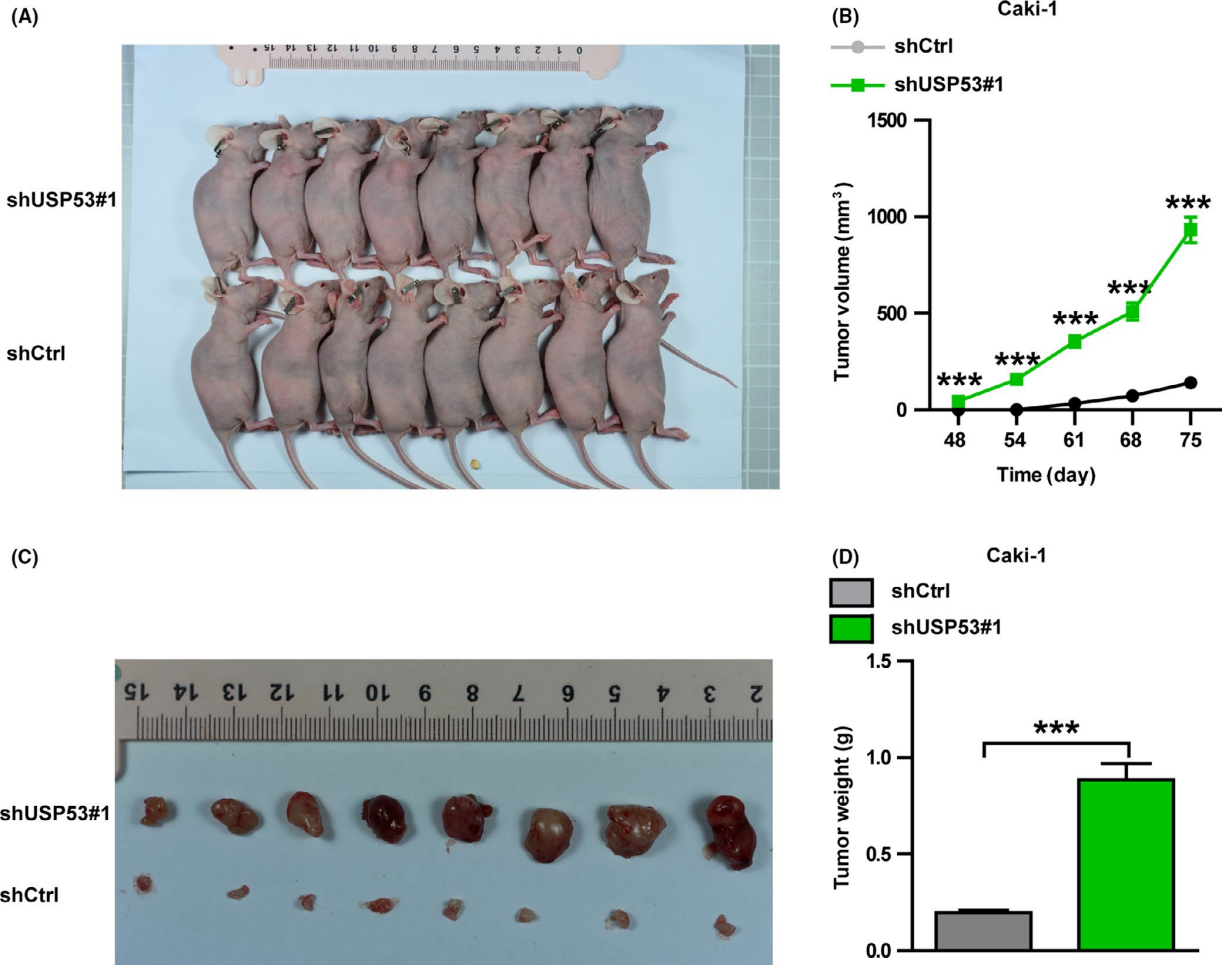
Knockdown USP53 promotes the growth of renal cancer cells in vivo. (A) Representative images of subcutaneous tumor formation in nude mice derived from USP53‐depleted Caki‐1 cells and control cells 75 days after subcutaneous injection. (B) Tumor volumes were measured and statistically analyzed between Caki‐1 control and USP53‐depleted cells. (C) Nude mice were sacrificed and tumors were removed for photographing. (D) The isolated tumors were weighed. Representative images and quantification are shown (*p < 0.05, **p < 0.01, ***p < 0.001). Data are shown as mean ± SEM

The proliferation and metastasis of ccRCC were significantly inhibited by USP53 *in vitro*. To detect if USP53 plays the same role in the *in vivo* environment, we knock down USP53 expression in Caki‐1 cells via shRNA and then tested by xenograft experiments. Caki‐1 tumor cells after USP53 knockdown were transplanted subcutaneously on the right side of the scapula of nude mice. After implantation, nude mice were detected for tumor formation on day 40, and they were sacrificed on day 75 (Figure 7A). Tumor volumes were measured once a week (Figure 7B), after mice were killed and tumors were excised and photographed (Figure 7C). We weigh isolated tumors and make statistics and we found the tumor weight after USP53 knockdown is much larger than shCtrl (Figure 7D). The results show that knockdown of USP53 significantly promotes tumor growth of ccRCC *in vivo*.

In summary, our various *in vivo* and *in vitro* experiments prove that USP53 regulates the occurrence and development of ccRCC through the NF‐κB pathway.

## 4 DISCUSSION

Renal cancer is one of the most deadly cancers in the world due to difficult diagnosis and poor prognosis. Current therapies are difficult to treat metastatic kidney cancer, which is the leading cause of death in patients with kidney cancer and therefore urges the development of new targets for prevention and treatment. In our study, we present a novel oncogene USP53 during ccRCC progression, in which USP53 expression decreases with increasing malignancy of ccRCC, regulating the NF‐κB signaling pathway to inhibit ccRCC proliferation and metastasis, thereby demonstrating that it functions as a potential therapeutic target in the tumorigenesis of ccRCC.

In the first, bioinformatic data mining found that USP53 may be involved in ccRCC. We compared four GSE databases of ccRCC and found that USP53 expression in tumor tissue was significantly lower than normal tissue and we also got the same result in TCGA‐KIRC database. In‐depth analysis of TCGA database clinical sample pathological diagnosis grading information show analysis of USP53 expression is the correlation with tumor malignancy. We then analyzed the correlation between USP53 expression and overall survival of ccRCC using the TCGA database. The USP53 high expression population clearly exhibited a good prognosis and survival. Compared with normal kidney cells, USP53 is significantly lower expressed in kidney cancer cells. These data suggest that USP53 may act as an oncogene in kidney cancer. Functionally, ectopic USP53 expression reduced cell proliferation and cell migration *in vitro* and vice versa. Downregulation of USP53 also promote cell proliferation *in vivo*. RNA sequencing shows overexpression of USP53 leads to decreased expression of a large number of inflammatory genes. USP53 overexpression decreased the phosphorylation of IKKβ and P65 and increase the expression of IκBα. USP53 enhances the stability of IκBα by deubiquitinating IκBα, thereby inhibiting the activity of the NF‐κB pathway. We described a new mechanism by which USP53 inhibits ccRCC survival via the downregulation of the NF‐κB signaling pathway. Our findings not only provide a novel insight into the molecular regulation of ccRCC, but also highlight USP53 as a potential target in ccRCC development.

USP53 was shown to be associated with pediatric cholestatic liver disease, obese, cantu syndrome, and mice progressive hearing loss.^30^, ^31^, ^32^, ^33^ However, there are no reports on the specific molecular mechanism of USP53 in cancer. We showed that USP53 is negatively correlated with ccRCC tumor progression and survival prognosis. Our data indicate that USP53 inhibits cancer cell proliferation and migration. Many genes that are related with NF‐κB signaling pathway are downregulated after USP53 overexpression. NF‐κB regulates cellular inflammatory responses in a variety of ways, and these inflammatory responses are essential in the development of tumors.^34^, ^35^ Many molecules are phosphorylated and ubiquitinated to regulate cell inflammation in NF‐κB pathway.^34^, ^36^ USP53 mediate NF‐κB signaling pathway is also used for verification in other tumor contexts. The regulation mechanism of USP53 mediated NF‐κB signaling pathway still needs to be explored then ascertain the functional effects of USP53 in ccRCC. Here, we can guess that USP53 may inhibit the NF‐κB pathway by inhibiting the degradation of IκBα. In summary, we demonstrated that the USP53 gene is a new suppressor of ccRCC, and that its function is regulated by the NF‐κB signaling pathway. Further understanding of USP53 regulation in ccRCC may assist the development of new therapeutic strategies for targeted tumor therapy.

## 5 CONCLUSION

In summary, our data showed that USP53 inhibits ccRCC proliferation and metastasis through NF‐κB pathway inactivation. The overexpression of USP53 is accompanied by changes in many inflammatory genes in the NF‐κB​ pathway. USP53 inhibits the p65 and p50 complex by deubiquitinating IκBα, and further inhibits the activity of the NF‐κB pathway. These findings may help better understand the pathogenesis of ccRCC and introduce new potential therapeutic targets for kidney cancer patients.

## AUTHOR CONTRIBUTIONS

Dingwen Gui and Zhufeng Dong contributed equally to this work. All authors contributed to the study conception and design. Material preparation, data collection, and analysis were performed by ZD and DG. The first draft of the manuscript was written by ZD and DG. All authors commented on previous versions of the manuscript. All authors read and approved the final manuscript.

## ETHICAL APPROVAL

All animal studies were carried out in accordance with the Animal Care and Use Committee of Wuhan University, which is consistent with the Association for Assessment and Accreditation of Laboratory Animal Care international (AAALAS) guidelines.

## Data Availability

All the data that support the findings of this study are available from the corresponding author upon reasonable request.
